# Global assembly of microbial communities

**DOI:** 10.1128/msystems.01289-22

**Published:** 2023-05-17

**Authors:** Jianing Wang, Zhuo Pan, Jianshui Yu, Zheng Zhang, Yue-zhong Li

**Affiliations:** 1 State Key Laboratory of Microbial Technology, Institute of Microbial Technology, Shandong University, Qingdao, China; CNRS Delegation Alpes, Lyon, France

**Keywords:** microbial community assembly, earth microbiome project, deterministic and stochastic processes, community-internal influences, source track, core microorganisms

## Abstract

Different habitats harbor different microbial communities with elusive assembly mechanisms. This study comprehensively investigated the global assembly mechanisms of microbial communities and effects of community-internal influencing factors using the Earth Microbiome Project (EMP) data set. We found that deterministic and stochastic processes contribute approximately equally to global microbial community assembly, and, specifically, deterministic processes generally play a major role in free-living and plant-associated (but not plant corpus) environments, while stochastic processes are the major contributor in animal-associated environments. In contrast with the assembly of microorganisms, the assembly of functional genes, predicted from PICRUSt, is mainly attributed to deterministic processes in all microbial communities. The sink and source microbial communities are normally assembled using similar mechanisms, and the core microorganisms are specific to different environment types. On a global scale, deterministic processes are positively related to the community alpha diversity, microbial interaction degree and bacterial predatory-specific gene abundance. Our analysis provides a panoramic picture and regularities of global and environment-typical microbial community assemblies.

**IMPORTANCE:**

With the development of sequencing technologies, the research topic of microbial ecology has evolved from the analysis of community composition to community assembly, including the relative contribution of deterministic and stochastic processes for the formation and maintenance of community diversity. Many studies have reported the microbial assembly mechanisms in various habitats, but the assembly regularities of global microbial communities remain unknown. In this study, we analyzed the EMP data set using a combined pipeline to explore the assembly mechanisms of global microbial communities, microbial sources to construct communities, core microbes in different environment types, and community-internal factors influencing assembly. The results provide a panoramic picture and rules of global and environment-typical microbial community assemblies, which enhances our understandings of the mechanisms globally controlling community diversity and species coexistence.

## INTRODUCTION

Thanks to the booming sequencing technologies, microbial community compositions have been extensively described in various habitats over the past 2 decades. In general, different habitats harbor different microbial communities, but the mechanisms for community assembly remain controversial. The niche-based theory hypothesizes the governing of deterministic factors such as species traits, interspecies interactions and environmental filtering on the community structure (1), while the neutral theory assumes that the community structures are independent of species traits and governed by stochastic processes of birth, death, colonization, extinction, and speciation (2). The deterministic processes contain the heterogeneous selection (also called variable selection) and the homogeneous selection subprocesses, while the stochastic processes are divided into three aspects: dispersal limitation, homogenizing dispersal, and drift. Heterogeneous selection causes communities to be more dissimilar while homogeneous selection makes communities more similar. Of the stochastic subprocesses, dispersal limitation leads to more dissimilar structures, homogenizing dispersal homogenizes communities, and drift signifies community composition random changes due to the inherent birth, death, and reproduction of microorganisms.

Ofiteru et al. once combined the deterministic and stochastic processes to explain the assembly of a microbial wastewater treatment community (3). The contributions of the assembly processes and subprocesses to microbial communities could be quantified based on the null model (4, 5), thus providing a possibility to analyze their relative importance in governing the construction of microbial communities in different habitats. The assembly processes of microbial communities have been explored in diverse environments, including soils, waters, sediments, plant rhizospheres and leaf surfaces, animal guts and surfaces. Extensive investigations have shown that similar ecosystems often obtained similar assembly mechanisms for microbial communities but sometimes received different conclusions; for example, deterministic processes were revealed to be dominant for bacterial community assembly in sediments of the Qinhuai River (6), the Yangtze River (7), the Thames (8), the Yellow River estuary, (9) and the Qiantang River estuary (Hangzhou Bay) (10). However, the marine prokaryotic community assembly was shown to be mainly controlled by stochastic processes based on Malaspina-2010 and Tara-Oceans data (11) or dominated by the homogeneous selection of deterministic processes in the South Pacific Gyre (12). Different conclusions were also obtained for the archaeal community assembly in sediments of the eastern Chinese marginal seas (13, 14). Although community assembly of crop-associated fungi is strongly influenced by deterministic selection exerted by the plant host, Gao et al. revealed that stochastic forces (drift or stochastic dispersal) were acted on mycobiome assembly in sorghum leaves and roots early in host development and drought stressed (15). Thus, the microbial community assembly is sophisticated but probably has some intrinsic regularities.

Wu et al. once systematically sampled sludges collected from 269 wastewater treatment plants on six continents (16). With the global data set, the authors revealed that the activated sludge microbiomes can be spatially turned over, which is scale-dependent, i.e., community similarity decreases as the geographical distance increases, and the turnover is largely driven by stochastic processes (dispersal and drift), accompanied with important contributions from deterministic factors (temperature and organic input). Similarly, Clarke et al. showed that the bacterial epibiont communities on Antarctic krill exhibit spatial structuring, driven mainly by distance rather than environmental factors, especially for strongly krill-associated bacteria (17). In addition to the influence of geographical distance and environmental factors, some community-internal factors were found to function on community assembly, such as the total organic carbon metabolism and mineralization potential (18), the microbial diversity and community ecology function (19).

The Earth Microbiome Project (EMP) was founded in 2010 to globally sample microbial communities by scientific crowdsourcing and standardized methods (20). Based on the data, some momentous insights into microbial ecology have been obtained on a global scale. For example, Shoemaker et al. tested 14,962 samples from the EMP and other data and demonstrated a lognormal dynamic for the distribution of microbial abundance and diversity–abundance scaling laws (21). Walters and Martiny analyzed the alpha-, beta-, and gamma-diversities of bacterial assemblages based on 11,680 samples compiled by the EMP and found the importance of spatial environmental heterogeneity in driving bacterial diversity (22). Similarly, the sequenced proportion of global prokaryotic genomes (23) and a global microbial co-occurrence network (24) were also assessed based on the EMP data.

**Fig 1:**
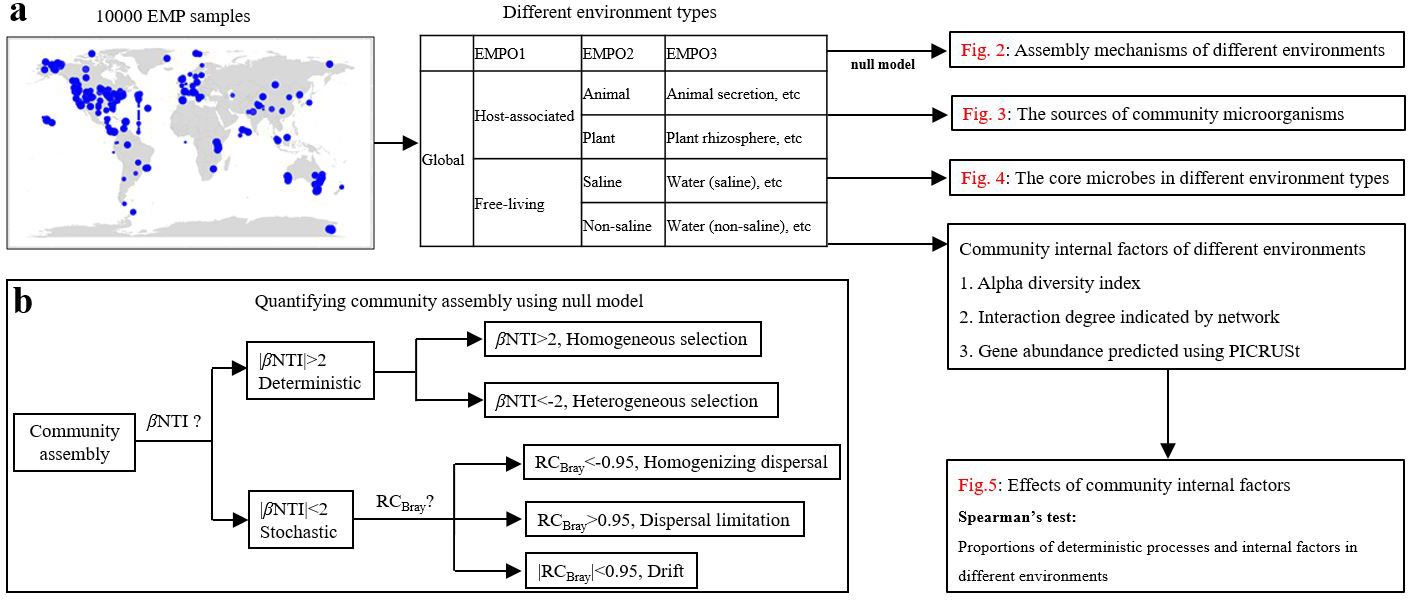
Schematic diagram of the analysis method of this study. (a) Random sampling from the EMP samples with different environmental factors or under different environment types. Community assembly mechanisms of different environments using the null model (Fig. 2), the sources of community microorganisms (Fig. 3), the core microbes in different environment types (Fig. 4), the effects of community internal factors on global microbial community assembly (Fig. 5). (b) Analysis process of microbial community assembly mechanisms using the framework developed by Stegen et al. (4, 5). EMP, Earth Microbiome Project; βNTI, β nearest-taxon index; RC_bray_ Raup–Crick based on Bray-Curtis.

In this study, we attempted to determine the regularities of microbial community assembly on a global scale and environment-type scales based on the EMP data set. We established a pipeline combined with various approaches to investigate quantificational contributions of the deterministic and stochastic processes for microbial community assembly, the sources of community microorganisms using Sourcetracker, the core microbes in different environment types based on occurrence rate and relative abundance, and the community-internal influencing factors with Spearman’s test (Fig. 1). This comprehensive investigation exhibited a panoramic picture and some regularities of global microbial community assemblies.

## MATERIALS AND METHODS

### Data collection

The data used in this study are all from the EMP. In detail, an amplicon sequence variant (ASV) table (emp_deblur_90 bp.subset_10 k.rare_5000.biom) containing 10,000 globally collected samples, a total of 262,011 ASVs and their abundance and nucleic acid sequence information was acquired from: http://ftp.microbio.me/emp/release1/otu_tables/deblur/. The functional genes profiles (cog_predictions.biom and ko_predictions.biom) associated with the ASVs based on the Phylogenetic Investigation of Communities by Reconstruction of Unobserved States (PICRUSt) (v. 1.1.4) program (25) were acquired from: http://ftp.microbio.me/emp/release1/otu_tables/picrust/. Sample environmental information and alpha diversity indices (emp_qiime_mapping_subset_10 k.tsv) were obtained from: http://ftp.microbio.me/emp/release1/mapping_files/.

### Core ASVs and taxonomic annotation

The core ASVs were defined to meet the following two conditions: mean relative abundance greater than 0.1% and occurrence in more than half of the samples. The taxonomy of each core ASV was annotated based on the Ribosomal Database Project (RDP) database (rdp_16 s_v16_sp.fa) (26) using USEARCH (v. 10.0.240) (27) at the threshold of 80% confidence. The alignment between the 24 core ASVs of soil (nonsaline) in this study and the 511 core ASVs of global soil bacteria (28) was performed using BLASTn (v. 2.10.0).

### Quantification of community assembly processes

To explore the assembly mechanisms of microbial communities, we constructed subgroups from the EMP samples by random sampling (Fig. 1a). A subgroup contained 40 random samples (each containing 5,000 ASV sequences), and the produced 200,000 ASV sequences were constructed into the sub-ASV table by using the “qiime feature-table filter-samples” program in QIIME 2 (v. 2020.2) (29). Phylogenetic trees of the representative ASV sequences were constructed using the “qiime phylogeny align-to-tree-mafft-fasttree” program. We repeated the above random sampling for 50 times at different EMPO (EMP ontology) levels, thus obtained 50 sub-ASV tables and their corresponding phylogenetic trees, which were used as input files to quantify community assembly processes.

A framework developed by stegen et al. was used to analyze the ecological processes for community assembly (4, 5, 30). This framework quantifies ecological processes based on both phylogenetic (β nearest-taxon index; βNTI) and taxonomic diversity (Raup‒Crick based on Bray-Curtis; RC_bray_). In detail, β mean nearest-taxon distance (βMNTD) was used to quantify turnover in the phylogenetic structure of communities; abundance-weighted βMNTD was calculated using the comdistnt function in the picante (v. 1.8.2) package. Next, a between-community null modeling approach (999 randomizations) was applied to infer community assembly processes by calculating the βNTI, which represents the deviation between the observed βMNTD and the expected. As the expected βMNTD represents the dominance of stochastic processes, the value of βNTI can be used to infer the dominance of stochastic and deterministic processes. RC_bray_ is also based on a null model test of the Bray‒Curtis taxonomic β-diversity index. The βNTI values and RC_bray_ were combined to estimate the relative contribution of homogeneous selection, heterogeneous selection as well as dispersal limitation, homogenizing dispersal and drift in community assembly. βNTI < −2 or > +2 indicates homogeneous selection or heterogeneous selection. RC_bray_ < −0.95 or > +0.95 indicates significant deviations from the null model expectation. |βNTI| < 2 with RC_bray_ <−0.95 or > +0.95 suggested that the deviation was contributed by homogenizing dispersal or dispersal limitation; when |βNTI| < 2 and |RC_bray_| <0.95, the shift of community composition was from drift (Fig. 1b).

Effects of deterministic or stochastic processes on gene assembly were performed using the unweighted Raup–Crick index (31, 32) or weighted Roup-Crick index based on Bray–Curtis (4) described as previous study.

### Source tracking and co-occurrence network

Source tracking was performed with Sourcetracker (v. 1.0.1) in R (v. 3.6.3), which uses a Bayesian approach to estimate the proportion of contaminants in a given community (referred as sink) that comes from a potential source environment (referred as source) (33). We selected the top 500 abundant ASVs of each environment type and performed pairwise calculations of the Spearman’s r and *P* values that are associated with the relative abundance using the psych (v. 2.0.9) package in R. Values of |Spearman’s r| > 0.5 and *P* < 0.05 indicated valid relationships. The network topological features were calculated using Gephi (v. 0.10.1).

### Correlation between community assembly processes and influencing factors

To explore the relationships between community assembly and community-internal factors, the Spearman’s tests were conducted between the proportion of deterministic processes in each of 16 environment types and the alpha diversity indices, or the degree of microbial interaction and the relative abundance of specific genes according to reported methods (9, 19).

## RESULTS

### Microorganism and gene assemblies in global microbial communities

We analyzed the assembly processes of global and environment-typical microbial communities using EMP data set (details are shown in the Methods section) based on a quantitative framework (4, 5). This framework quantifies ecological processes from both phylogenetic and taxonomic diversity (βNTI and RC_bray_) based on the null model test. βNTI is calculated from the phylogenetic diversity index βMNTD, and RC_bray_ is from the Bray‒Curtis taxonomic β-diversity index (Fig. 1b). The EMP hierarchically classifies the samples of different environments at three levels (EMP ontology, and EMPO): free-living and host-associated EMPO1, saline and non-saline of the free-living plant and animal of the host-associated EMPO2, and all in a subdivision of 17 environment types (EMPO3) (20). As the pretesting results show, the sampling size from 30 to 50 in a subgroup (10 sampling times) produced similar proportion results of the deterministic and stochastic processes with no significant difference (*P* > 0.3) (Fig. S1), and the proportion of deterministic processes tended to be stable after approximately 30 sampling times with the sampling size of 40 (Fig. S2). Accordingly, we constructed 50 subgroups each containing 40 random samples for the calculation of βNTI and RC_bray_ at each EMPO level and each environment type (details refer to the Methods section). For reliability confirmation, the proportion changes of deterministic processes along with different sampling times at different levels are provided in Fig. S3.

**Fig 2:**
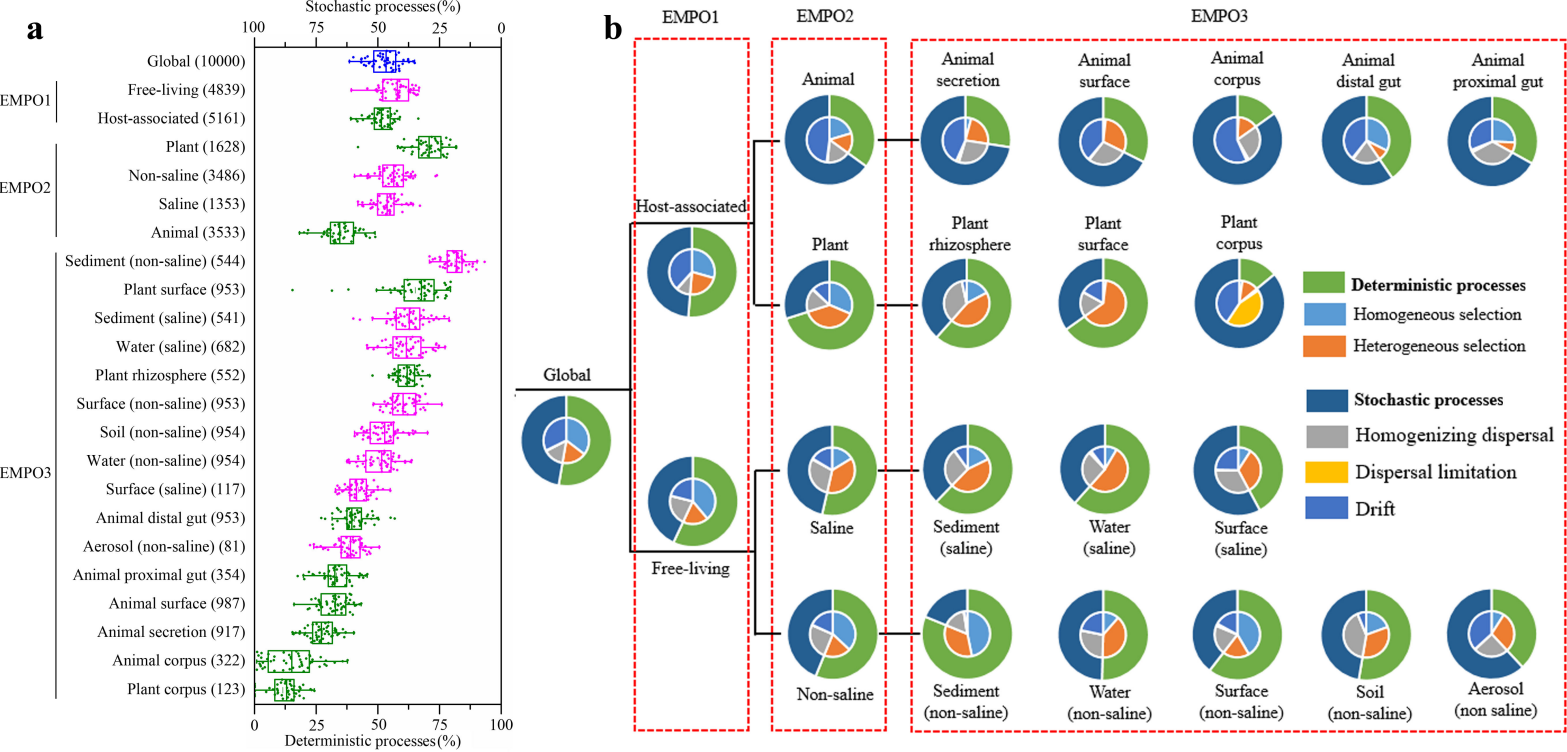
Assembly mechanisms of global microbial communities. (a) The proportions of deterministic and stochastic processes of microbial communities in different types of environments. Each point represents the result of a single random sampling. For the box plots, the middle line indicates the median, the box represents the 25th–75th percentiles. Environment types were classified by EMPO (EMP ontology), blue represents the global, olive represents host-associated and magenta represents free-living. The number of samples in the corresponding environment is shown in parentheses. (b) Assembly mechanisms within the deterministic and stochastic processes for microbial communities in different environment types.

On a global scale, deterministic processes occupied 52.70% ± 6.32% of the microbial community assembly processes, which was slightly higher than the proportion of stochastic processes (47.30% ± 6.32%) (Fig. 2a). This result indicated that the assembly of microbial communities on our planet was controlled in a rather equal ratio of the deterministic and stochastic processes. Specifically, the proportion of deterministic processes in free-living samples (56.70% ± 6.00%) was significantly higher than that in host-associated samples (51.30% ± 5.29%) (*P* < 0.01). At the EMPO2 level, the proportions of deterministic processes in saline and non-saline free-living microbial communities were 53.57% ± 5.51% and 56.16% ± 6.81%, respectively, with no significant difference (*P* > 0.05). Surprisingly, the proportions of deterministic processes were distinct in the microbial communities associated with animals (34.99% ± 6.97%) and plants (70.11% ± 7.45%) (*P* < 0.01). Furthermore, at the EMPO3 level (hypersaline containing only 13 samples was excluded), the proportions of deterministic processes ranged from 81.19% ± 4.92% of sediment (non-saline) to 11.50% ± 6.79% of plant corpus. In general, the deterministic processes played a major role in both the free-living samples (except aerosol (non-saline) and surface (saline)) and the plant-associated samples (except plant corpus), while the stochastic processes were the major contributor for the microbial community assembly in all the animal-associated samples.

Functional genes are critical for microorganisms to survive and to perform their ecological functions in environment. Like the community microorganisms, genes in communities are assembled biogeographically (31, 34). We further analyzed gene assembly of the global communities. The gene profiles were predicted from the PICRUSt(25) based on the global environmental ASV sequences. Totally, we revealed 6,909 functional genes annotated by Kyoto Encyclopedia of Genes and Genomes (KEGG) or 4,792 functional genes annotated by Clusters of Orthologous Groups (COG). The gene assembly in 16 environment types (excluding the hypersaline type) was analyzed using an unweighted model-based method (refer to the Methods section) (31, 32). We found that, different from the varied relative importance of the deterministic and stochastic processes for the microorganism assembly, the gene assembly was always mainly attributed to the deterministic processes in microbial communities, ranging from 60.86% ± 2.86% (surface (saline)) to 94.18 % ± 1.08% (plant corpus) based on the KEGG annotation, or from 55.21% ± 3.39% (water (saline)) to 92.34% ± 1.37% (plant corpus) based on the COG annotation (Fig. S4). The analysis based on a weighted model showed similar results (Fig. S4). It is well known that many coexisting but taxonomically distinct microorganisms may encode the same metabolic functions, leading to functional redundancy (35). The gene assembly results provide a new evidence for the existence of functional redundancy, i.e., the genes in a microbial community are subject to the effects of deterministic selections because the same gene functions could be charged by different microbial taxa. Notably, the bacterial reference databases employed by PICRUSt have not been updated since 2013 and, thus, lack thousands of recently added gene families. However, these limitations have no significant effect on our analysis of the gene assembly, which is calculated by the gene distribution rather than their functions.

### Subprocess contributions for community similarity and dissimilarity

Both of the deterministic and stochastic processes contain subprocesses contributing to the similarity or dissimilarity characteristics of microbial communities (1, 2). As shown in Fig. 2b), for the global microbial community assembly, homogeneous selection (35.39% ± 6.18%) and drift (32.28% ± 6.33%) were the dominant subprocesses, heterogeneous selection (17.31% ± 3.71%) and homogenizing dispersal (14.61% ± 3.75%) contributed less. Thus, on a global scale, homogeneous selection, as well as homogenizing dispersal, contributed the similarity of microbial communities, while the variability of microbial birth, death, and reproduction, as well as the environment variable selection, made community diverse. For microorganisms, the dispersal limitation had a very weak effect on the global community assembly (0.42% ± 0.33%).

For the assembly of free-living microbial communities, homogeneous selection was the predominant contributor (38.87% ± 6.04%), while the assembly processes in saline and non-saline samples were controlled mainly by heterogeneous selection (37.3% ± 4.53%) and homogeneous selection (36.96% ± 7.73%), respectively (Fig. 2b). This result indicated that the heterogeneous and homogeneous selections had an opposite relative importance for the assembly of saline and non-saline microbial communities. For host-associated microbial communities, the dominant driver was drift (47.96% ± 7.78%) for the microbial assembly in animal-associated samples. However, the microbial assembly in plant-associated samples was attributed to both the heterogeneous (38.72% ± 6.79%) and homogeneous (31.39% ± 5.86%) selection processes. Thus, the major driving mechanisms varied for the similarity and dissimilarity of microbial communities associated with hosts or free-living, saline or non-saline, and animals or plants.

At the EMPO3 level, drift, homogenizing dispersal, and heterogeneous selection were generally the dominant mechanisms for the community compositions of host-associated microbiomes, except that homogeneous selection was more important than heterogeneous selection in animal proximal and distal guts, and that drift contributed little (3.32% ± 1.5%) to the plant rhizosphere. Among the 16 environment types (except hypersaline), dispersal limitation was predominant only in plant corpus-associated samples (43.79% ± 5.51%), and rarely functioned in any other environment, from 0.08% ± 0.13% in soil (non-saline) to 2.04% ± 1.37% in animal secretion (Fig. 2b). Similar to that of the host-associated samples, heterogeneous selection and homogenizing dispersal were also often the major contributors for free-living microbial community assembly, but homogeneous selection played a more important role than heterogeneous selection in sediment (non-saline) and surface (non-saline) samples. Among the EMPO3 environments, the assembly of non-saline microbial communities normally had the most diverse ecological processes. The above analysis results suggested that microbial communities in the same environment types are generally assembled in similar processes, but using different mechanisms across different types, reflecting the influences from diverse environments.

Overall, of the three stochastic subprocesses, homogenizing dispersal, dispersal limitation and drift made the highest contributions in non-saline soil (41.05% ± 6.14%), plant corpus (43.79% ± 5.51%), and animal corpus (56.65% ± 9.21%), respectively. High contribution of homogenizing dispersal in soil (non-saline) probably reflected the frequent interflows of soil microbial communities with others, while high contributions of dispersal limitation in plant corpus and drift in animal corpus suggested the decreased selection pressure from hosts and rich nutrients, leading to a decrease of directed selection and filtering on microbial communities. Of the two deterministic subprocesses, the highest contributions for the assembly of microbial communities appeared in sediment (non-saline) (homogeneous selection, 46.78% ± 3.75%) and plant surface (heterogeneous selection, 63.1% ± 11.78%). High contribution of homogeneous selection in sediment (non-saline) suggested their similar environmental conditions, while the high contribution of heterogeneous selection in plant surface probably reflected specific selection by different plants.

### Sources of community microorganisms in different environment types

**Fig 3:**
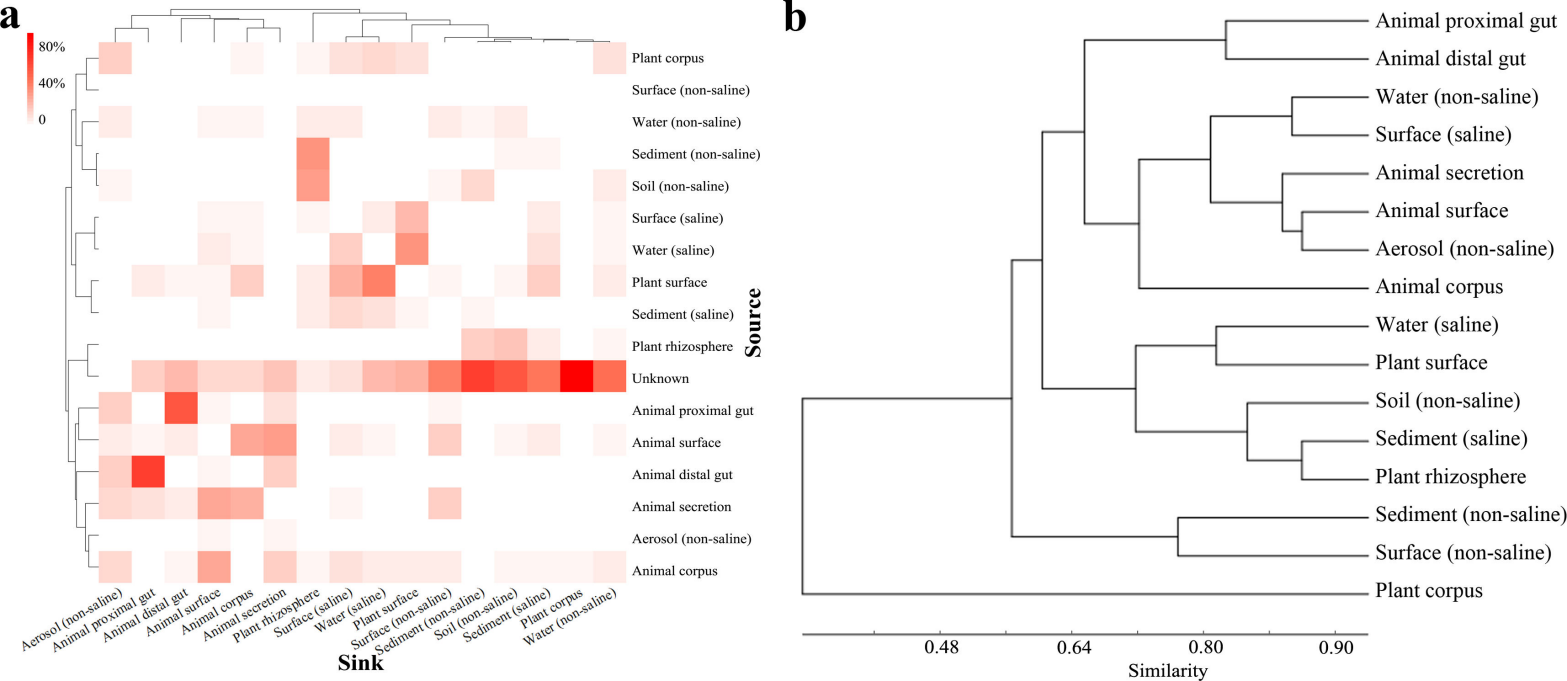
The sources of community microorganisms in different environment types (EMP ontology, EMPO3). (a) Results of the source tracking for microbiota in different environment types (EMPO3). **“**Sink” represents the environment type analyzed, and “source” shows the proportion of microbial sources from other environments of the sink. (b) Hierarchical clustering tree based on the Bray–Curtis metric using the ratio of assembly subprocesses (heterogeneous selection, homogeneous selection, dispersal limitation, homogenizing dispersal, and drift).

The EMP samples were globally collected from various environments. Our above analysis showed that dispersal limitation was rarely a limiting process for microbial community assembly in different environments, except the plant corpus-associated samples, suggesting the rationality to analyze the source of community microorganisms on a global scale. SourceTracker is a Bayesian approach to estimating the proportions of a community that comes from a set of source environments (33). Employing the method, Wu et al. suggested that the bacterial sources in sludge microbial communities collected from global wastewater treatment plants were majorly attributed from the EMP freshwater (46% on average), followed by soil (17%) and ocean (12%) environments (16). We similarly attempted to track the sources of microorganisms in different environment types on a global scale using the SourceTracker. We found that microorganisms in aerosol (non-saline) were mainly from the animal-associated microbial communities (totally > 59%) (Fig. 3a), suggesting the close exchange of movable animals with the aerosol. Notably, animal-associated microorganisms were mainly derived from animal-associated environments. For example, microorganisms in animal distal gut were mainly from animal proximal gut (49.4%), while microorganisms in animal surface microorganisms were mainly from animal corpus (24.2%) and animal secretion (25.9%). The plant rhizosphere microorganisms were mainly from non-saline sediment (29.1%) and non-saline soil (27.3%).

Using the assembly subprocesses in Fig. 2b, we performed a clustering analysis (based on the Bray–Curtis distance) and found that the assembly mechanisms of the sink community were generally similar to those of its major source community (Fig. 3b). For example, microorganisms in animal distal gut and animal proximal gut were sourced to each other, and the assembly mechanisms in these two environment types were rather similar, compared to those in other environment types. The source-tracking results suggested that frequent exchanging environments normally had similar assembly processes. Notably, the community assembly and source tracking are two independent analysis procedures, the former is generated from βNTI and RC_bray_ while the latter is based on the Bayesian analysis, the correlations between their results suggested some intrinsic roles inside.

We noticed that most of the environment types harbored more than 10% of the community microbiota with unknown sources (Fig. 3a; for details, refer to Table S1). The highest percentage appeared in the environment of plant corpus (78.4%), followed by sediment (non-saline) (55.3%), soil (non-saline) (47.9%), and water (non-saline) (41.5%). In contrast, the percentage of unsourced microorganisms in aerosol (non-saline) was the lowest (2.2%), followed by plant rhizosphere (6.0%), surface (saline) (10.3%) and animal surface (10.6%). For the microbial communities in plant corpus that contained the highest unsourced microorganisms, all the known sources were lower than 5%, and the highest known source was from animal corpus (4.98%). Because of the high ratio of unknown resources for many environment types, especially those free-living environment types, microbial flows across different environments are still mostly unclear. Hermans et al. once suggested that within the EMP, multiple regions are grossly under-sampled and poorly represented (36). The large number of unsourced microorganisms also suggested that more samples are needed to explore in different environment types.

### The core microorganisms in different environment types

**Fig 4:**
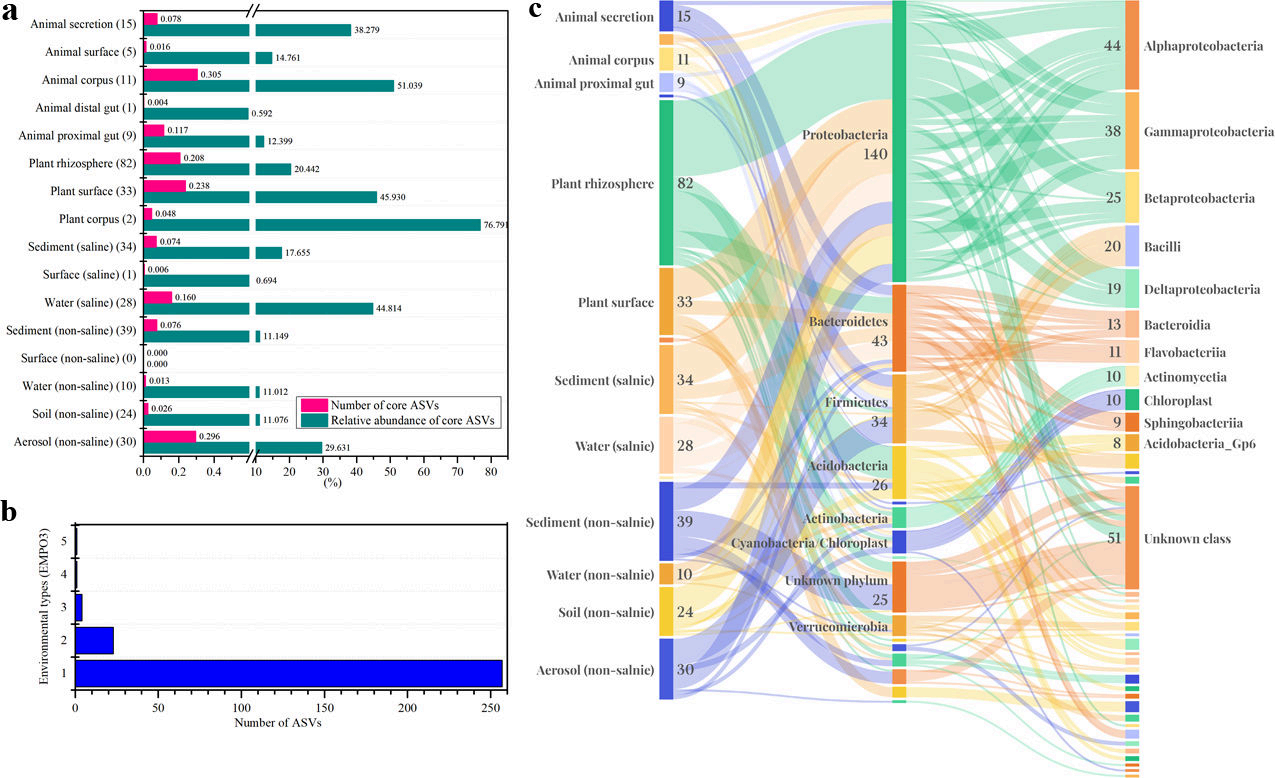
Number, relative abundance, and taxonomic composition of core ASVs under different environmental types (EMP ontology, EMPO3). (a) Percentage (number of ASV) and relative abundance (reads of ASV) of the core ASVs versus the remaining microbial ASVs under 16 environment types (EMPO3). The number of core ASVs in the corresponding environment is shown in parentheses. Core ASVs were selected based on: mean relative abundance >0.1% and existing in more than half of the samples under one environmental type. (b) Distribution of all core ASVs in the 16 environment types. (c) The taxonomic composition of the core ASVs at the phylum and class levels. ASV, amplicon sequence variant.

Microorganisms living in a community are the assembling results fitting for the environment and the community, and the core microorganisms in each environment are normally the key factors for community eco-functions. At the EMPO3 level, except the rarely sampled hypersaline environment, the total ASV numbers ranged from 3,604 of the animal corpus communities to 93,288 of the soil (non-saline) communities (Table S2). We defined the core ASVs as those appearing in more than half of the samples in an environment type with an average relative abundance of more than 0.1%. The results showed that the core ASVs varied greatly in different environment types, from 82 in plant rhizosphere to 0 in surface (non-saline) (Fig. 4a and Table S2). Compared to specific environment studies (16), the core ASV numbers were greatly limited in an environment type, primarily due to diversified subtype sources included. For example, the 953 samples of animal distal gut environment were collected from more than 90 kinds of different animals, and the human source (216) occupied only 22.7% of the total, which might be the reason for a single core ASV revealed in the animal distal gut samples.

The highest occurrence of core ASVs was in the animal corpus samples, containing 11 cores in the total 322 ASVs. However, although in a low ratio, the core ASVs were considerably high of their relative abundance. Among the 16 EMPO3 environment types, the relative abundance of core ASVs was higher than 10% in 13 environment types, higher than 30% in five environment types, and the highest reached 76.8% (plant corpus). We compared the 24 core ASVs of soil (non-saline) to the 511 core ASVs in global soil microbial communities (28) and revealed that 20 of the 24 core ASVs appeared in the 511 core ASVs with 100% identity. Thus, consistent with those of the global soil microbial communities (28) and the microbial communities of global waste treatment factories (16), although representing a small number, core ASVs normally occupied the majority of microbial communities in most global environment types.

Interestingly, of the total 286 core ASVs in 16 environment types, 257 occurred as core ASVs exclusively in a single environment type, 23 were the core ASVs in two environment types, and 6 in three or more environment types (Fig. 4b). The results indicated the environment-specificity of core ASVs. These core ASVs were taxonomically annotated into 14 phyla, and the *Proteobacteria* (140 ASVs), *Bacteroidetes* (43 ASVs) and *Firmicutes* (34 ASVs) were the three most popular phyla (Fig. 4c). At the class level, the core ASVs belonged to 37 classes, and *Alphaproteobacteria* (44 ASVs), *Gammaproteobacteria* (38 ASVs) and *Betaproteobacteria* (25 ASVs) were the three most popular classes. More details, including the taxonomic annotation information at lower levels, referred to Table S3.

### Influences of community-internal factors on the global assembly

**Fig 5:**
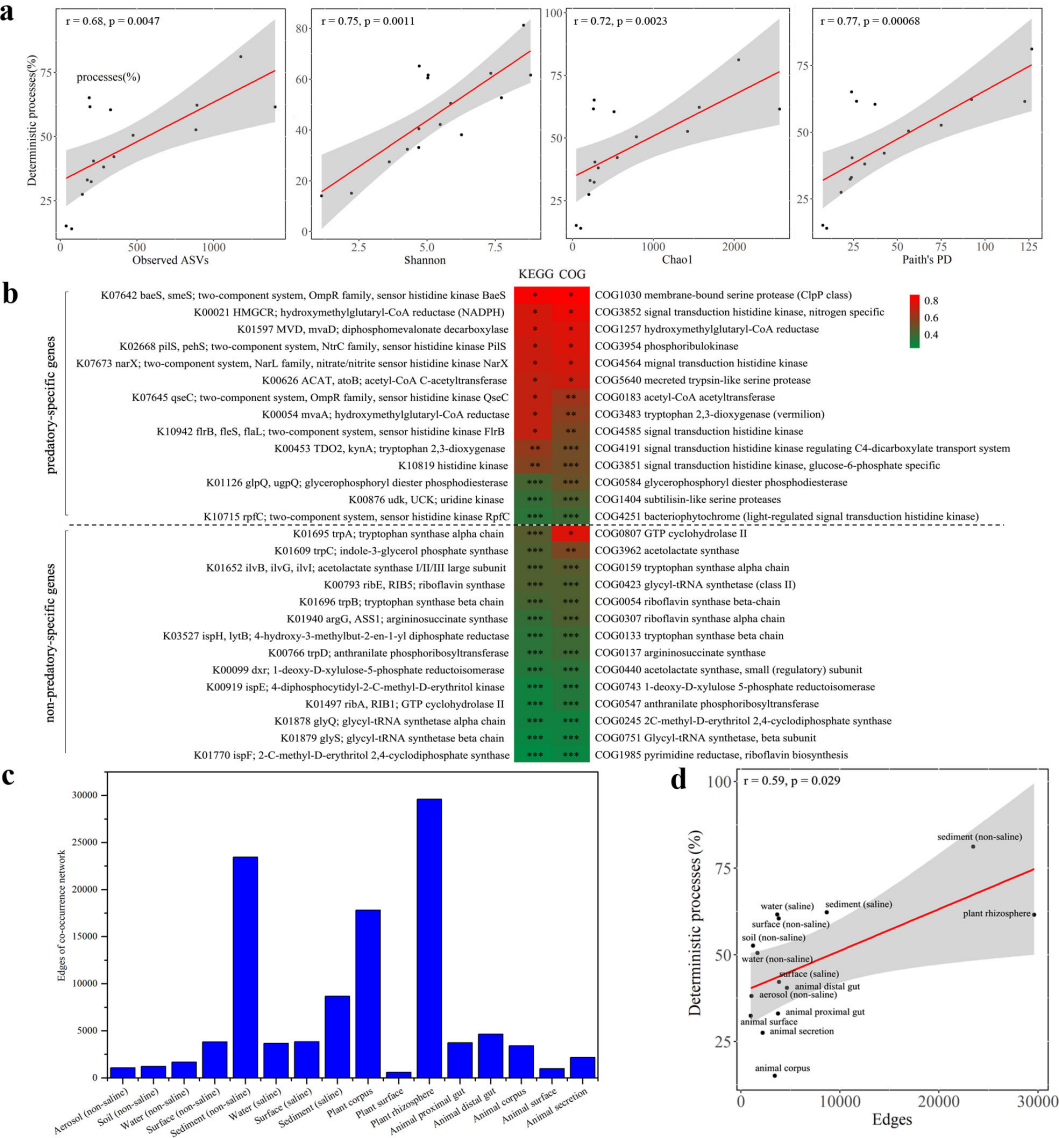
Effects of community internal factors on global microbial community assembly. (a) Relationships between the proportion of deterministic processes and alpha diversity indices (observed ASVs, Shannon, Chao1, and Faith’s PD) in 16 environment types (EMP ontology, EMPO3). (b) Relationships between the proportion of deterministic processes and the relative abundance of bacterial predatory-specific genes in 16 environment types (EMPO3) (*: *P* < 0.01, **: *P* < 0.05, ***: *P* > 0.05). (c) Edges of network in 16 environment types (EMPO3). (d) Relationships between the proportion of deterministic processes and degree of microbial interactions (edges of network), excluding plant corpus. ASV, amplicon sequence variant; Paith's PD, Faith's phylogenetic diversity; COG, Clusters of Orthologous Groups; KEGG, Kyoto Encyclopedia of Genes and Genomes; NADPH, nicotinamide adenine dinucleotide phosphate; MVD, diphosphomevalonate decarboxylase.

Microbial community assembly is controlled by both environment-external factors and community-internal factors. The EMP samples were collected from diverse environments with limited as well as no common environmental parameters. In this study, we investigated the effects of the community-internal factors on assembly, by analyzing the correlations between the assembly processes and the community-internal factors of alpha diversity, bacterial predatory-specific genes, and microbial interactions (refer to Fig. 1a). The Spearman’s r values of the deterministic processes were 0.68, 0.75, 0.72, and 0.77 for the observed ASVs, Shannon, Chao1, and Faith’s PD, respectively (Fig. 5a; *P* < 0.01). That is to say, the proportion of deterministic processes was positively correlated significantly with the alpha diversity indices of microbial communities. Consistently, Evans et al. also suggested, from their studies on microbial communities of leaf litter, the community with lower biomass and smaller population is more susceptible to drift (stochastic process) or founder effects (37). Thus, the alpha diversity is positively and negatively related to the deterministic processes and the stochastic processes, respectively.

Predatory bacteria, such as myxobacteria (38), are important controlling factors in microbial communities (39). Based on the gene profiles retrieved from the EMP observed ASVs using the PICRUSt program, we revealed that the abundance of bacterial predatory-specific genes rather than non-predatory-specific genes (40) was positively correlated with the proportion of deterministic processes across different environment types (Fig. 5b and Table S4). For genes annotated by KEGG, the Spearman’s r value was ranged from 0.3353 to 0.7912 for predatory-specific genes but ranged from 0.2176 to 0.4235 for non-predatory-specific genes. Similar results were also observed with COG genes (Fig. 5b and Table S4). This result suggested that the abundance of bacterial predatory-specific genes is positively correlated with the proportion of deterministic processes.

Interactions among microorganisms, which could be predicted using network inferences (41), are complicated and are also an important factor for community assembly. To analyze microbial interactions in different environment types, we selected the top 500 ASVs from each of 16 EMPO3 habitats to construct their interaction networks. The total relative abundance of the 500 ASVs in different environment types was ranged from 50.43% in sediment (non-saline) to 98.43% in animal corpus, showing that these dominants occupied the majority of microbial community. The aggregation of microbial community network varied significantly among different habitats (Fig. S5; the topological parameters reflecting the aggregation degrees of network graph are shown in Table S5), suggesting significant differences of the interactions in these environment types. Specifically, plant rhizosphere showed the highest degree of network aggregation, followed by sediment (non-saline) and plant corpus, while plant surface, animal surface and aerosol (non-saline) had the lowest degrees of network aggregation (Fig. 5c exhibits the edges of networks in different environment types). In a microbial co-occurrence network, edges could be employed to represent the statistically significant association between nodes (|Spearman’s r| > 0.5 and *P* < 0.05 in this study), thus to quantitatively represent the microbial interaction degrees (24). The correlation analysis showed that the Spearman’s r value of the deterministic processes with the network edges was 0.59 (*P* <0.05) (Fig. 5d; the plant corpus was excluded due to abnormal value). Together with previous studies on the correlations between nutrition and microbial interactions (42, 43), we suggested a positive correlation between alpha diversity and microbial interactions and the positive correlation of determination processes with both of alpha diversity and interaction degree.

## DISCUSSION

Community assembly is the process by which indigenous and immigrated species colonize and interact to establish and maintain a local community. Previous studies with globally sampled sludges of wastewater treatment plants determined that microbial communities in these similar environments are assembled in similar processes, and turnover of microbiomes is largely driven by stochastic processes as well as important contributions from deterministic factors (16). In this study, using the EMP data set, we determined that microbial communities in the same environment types are generally assembled using similar mechanisms, but the assembling mechanisms vary across environment types. Corresponding to specific community assembly mechanisms in different environments, the core microorganisms exhibit a strong environment-type specificity. Interestingly, we found that microbial exchange and dispersal frequently occur between habitats that are closely related in logic, which often share similar community assembly mechanisms, such as animal distal gut and animal proximal gut, plant rhizosphere, and non-saline soil.

The deterministic and stochastic processes, as well as the subprocesses that lead to similarity and variability, contributed approximately equally to microbial community assembly on a global scale. We noticed that, for host-associated microbial communities, stochastic processes were the main driver for the assembly of animal-associated microbial communities, while deterministic processes were mainly for the plant associated. Host-associated microbial communities play fundamental roles in plant and animal nutrition, development, and immunity (44), and the community compositions are in return influenced by the components, genotypes, and transmission patterns of hosts (45, 46). The major contribution of stochastic processes to the animal-associated microbial communities means that the assembly here is largely independent of the traits of the host, and this is consistent with the observed variations of microbiomes associated with animals (47 - 49). The subprocesses involved in animal-associated assembly mainly include the chance loss of a microbial taxon (drift) and passive dispersal from environment to host or between hosts (homogenizing dispersal) but are less affected by dispersal limitation. The probable reason is that animal hosts are generally permissive, somewhat akin to a filter, and the microbial communities in individual animals at a specific time may be shaped by the processes that are independent of, or weakly influenced by host factors (50 - 52). However, the major contribution of stochastic processes does not mean the disavowal against the deterministic roles across animal-associated microbial communities.

For plant-associated microbial communities, except the plant corpus, assemblies are mainly controlled by the deterministic processes. In fact, many studies have reported similar bacterial communities assembled in plant-associated samples (53 - 55). The phylogenetic conservation of community compositions suggests that the plant-associated community assembly is rather a deterministic process, i.e., governed by structural principles.

Community diversity is considered an important factor in generating and sustaining the ecosystem and ecosystem function (56 - 58), and within-habitat heterogeneity is a driver for the community diversity (59, 60). For example, the habitat heterogeneity of free-living communities is generally higher than that of host-associated communities, and, thus, they have higher community diversity (61). Thus, the positive correlation between the proportion of deterministic processes and the community alpha diversity suggests a close relationship of microbial community assembly to habitat heterogeneity. Probably, high habitat heterogeneity provides more unique ecological niches, which help to reduce competition and preserve high community diversity (62). Furthermore, the long-standing ecological theory suggests that predation is a filtering and selection pressure for community (63). Consistently, the positive correlation between the proportion of deterministic processes and the relative abundance of bacterial predatory-specific genes in microbial communities also supports the theory. That is to say, high diversity drives high productivity (58), which increases predatory-mediated control in the food web, leading to energy disproportionately flowing to the predation trophic level, and functional responses of predators to shifts in prey resource availability (64). Similarly, the positive correlations between the microbial interaction degree and the deterministic processes imply the importance of microbial interaction species-specificity in community assembly and stability.

In summary, this study provides a global panoramic picture and general regularities of microbial community assemblies on the earth. The global microbial community assembly is controlled by combined deterministic and stochastic processes, and the ratio of these two processes varies greatly across environment types, but the gene assembly in all microbial communities is mainly attributed to the deterministic processes. We found that the assembly mechanisms of the sink community are generally similar to those of its major source community, and the core microorganisms are environment specific. The deterministic processes for the global microbial community assembly are positively correlated to the community alpha diversity, microbial interaction degree and bacterial predatory-specific gene abundance.

## Data Availability

The main data supporting the findings of this study are available within the article and in its supplemental material. The raw data sets are available from EMP (http://ftp.microbio.me/emp/release1/). PICRUSt (v. 1.1.4) is available at https://github.com/picrust/picrust. RDP database (rdp_16s_v16_sp.fa) is available at http://www.drive5.com/sintax http://www.drive5.com/sintax. USEARCH (v. 10.0.240) is available at http://www.drive5.com/sintax. BLASTn (v. 2.10.0) is available at https://ftp.ncbi.nlm.nih.gov/blast/executables/blast+/. QIIME 2 (v. 2020.2) is available at https://docs.qiime2.org/2020.11/. Sourcetracker (v. 1.0.1) is available at https://github.com/danknights/sourcetracker. R (v. 3.6.3) is available at https://cran.r-project.org/. Picante (v. 1.8.2) is available at https://cran.r-project.org/src/contrib/Archive/picante/. Psych (v. 2.0.9) is available at https://cran.r-project.org/src/contrib/Archive/psych/. Gephi (v. 0.10.1) is available at https://gephi.org/users/download/. All custom codes used in this study are available from GitHub (https://github.com/wangjianing0618/Global-Microbial-Community-Assembly).

## SUPPLEMENTAL MATERIAL

Fig. S1 msystems.01289-22-s0001.tif Estimate of the sampling size (different letters indicate *p* > 0.3).

Fig. S2 msystems.01289-22-s0002.tif Proportions of the deterministic processes for global microbial community assembly with the sampling times.

Fig. S3 msystems.01289-22-s0003.tif Proportions of the deterministic processes for microbial community assembly in different environment types with the sampling times. a, EMPO1. b, EMPO2. c, EMPO3. Olive represents host- associated and magenta represents free-living.

Fig. S4 msystems.01289-22-s0004.tif The proportions of deterministic processes of microbial genes predicted using PICRUSt in different environment types (EMPO3). a, KEGG (unweighted model). b, COG (unweighted model). c, KEGG (weighted model). d, COG (weighted model). Each point represents the result of a single random sampling. For the box plots, the middle line indicates the median, the box represents the 25th–75th percentiles. Environment types were classified by EMPO, olive represents host-associated and magenta represents free-living.

Fig. S5 msystems.01289-22-s0005.tif Co-occurrence network analysis of the dominant bacterial ASVs (top 500) in different environment types (EMPO3). Each node represents an ASV, and the line means a significant correlation between the two ASVs (Spearman’s test, *r* > 0.5, *p* < 0.05).

Table S1 msystems.01289-22-s0006.xlsx Detailed results of the source tracking for microbiota in different environment types (EMPO3).

Table S2 msystems.01289-22-s0007.xlsx Number and relative abundance of core ASVs under different environment types (EMPO3).

Table S3 msystems.01289-22-s0008.xlsx Sequence and taxonomy information of core ASVs under 16 environment types (EMPO3).

Table S4 msystems.01289-22-s0009.xlsx Detailed information of the correlation analysis between the proportion of deterministic processes and the relative abundance of bacterial predatory-specific genes in different environment types.

Table S5 msystems.01289-22-s0010.xlsx The main topological parameters of the co-occurrence networks established using the top 500 ASVs in different environment types.

ASM does not own the copyrights to Supplemental Material that may be linked to, or accessed through, an article. The authors have granted ASM a non-exclusive, world-wide license to publish the Supplemental Material files. Please contact the corresponding author directly for reuse.
