# Supplementary figures and images for: Global assembly of microbial communities
Source: mSystems. 2023 May 17;8(3):e01289-22. doi: 10.1128/msystems.01289-22 (PMC10308889; doi:10.1128/msystems.01289-22)

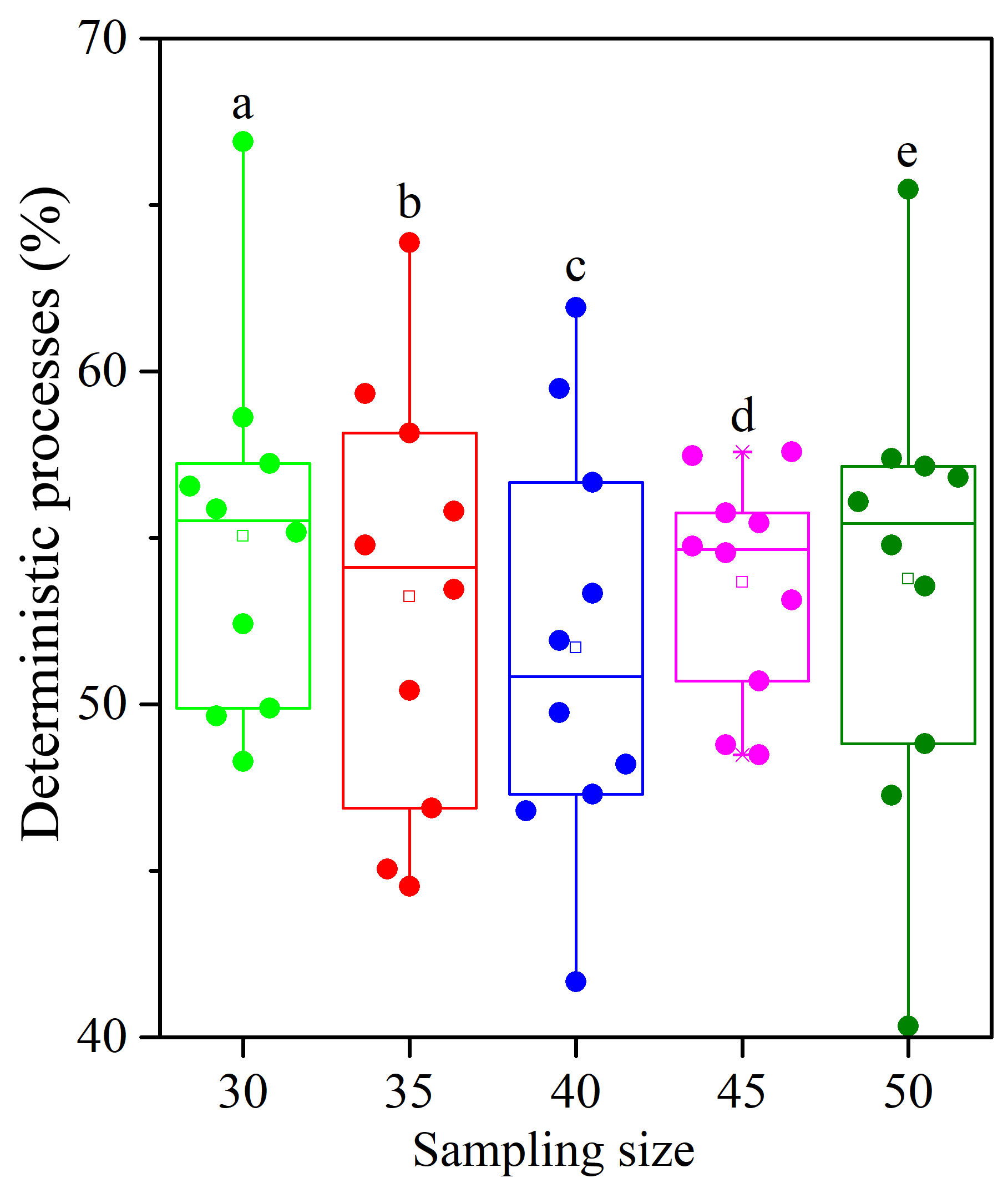

Supplement: Fig. S1 — Estimate of the sampling size (different letters indicate p > 0.3). [file msystems.01289-22-s0001.tif]

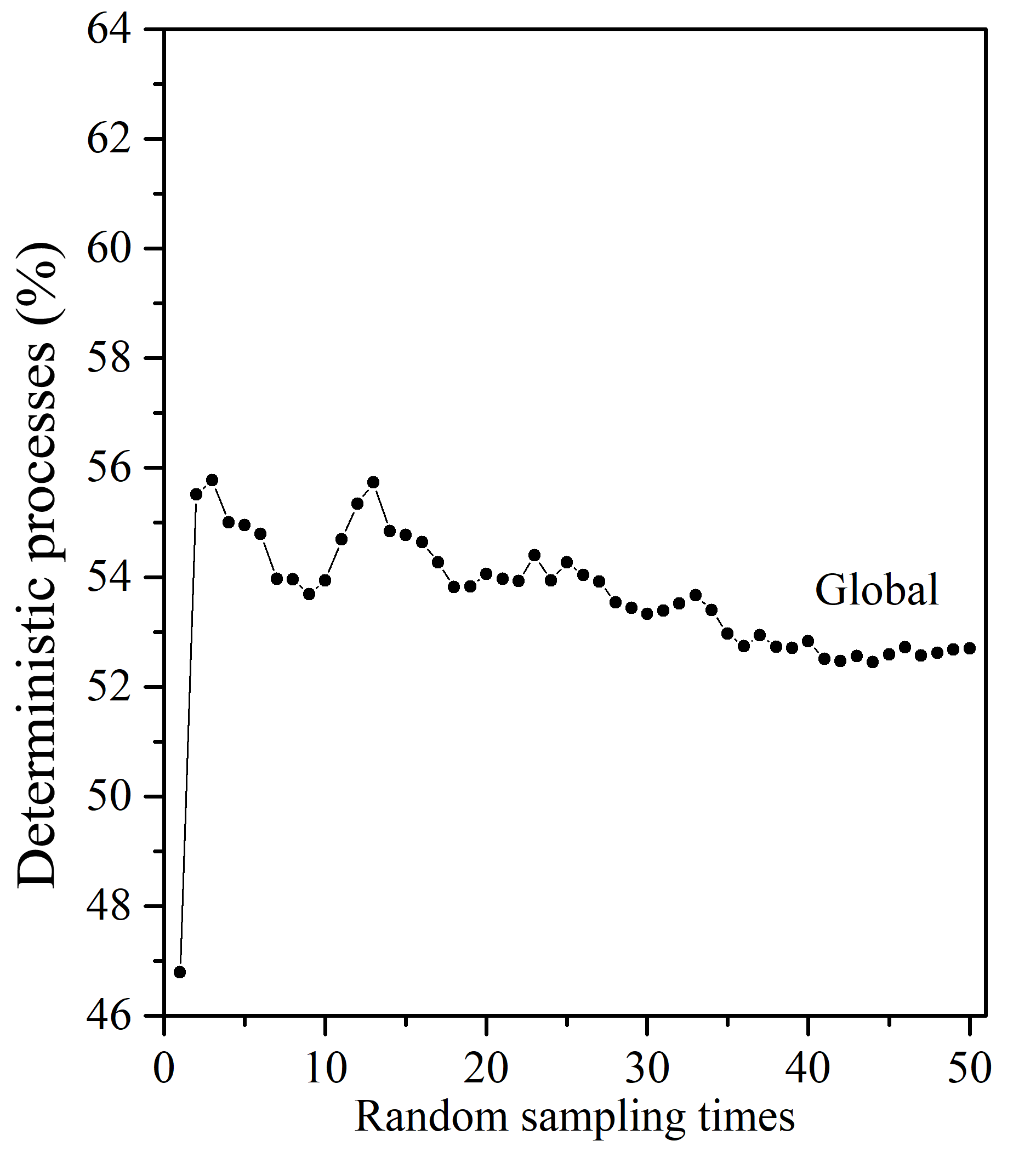

Supplement: Fig. S2 — Proportions of the deterministic processes for global microbial community assembly with the sampling times. [file msystems.01289-22-s0002.tif]

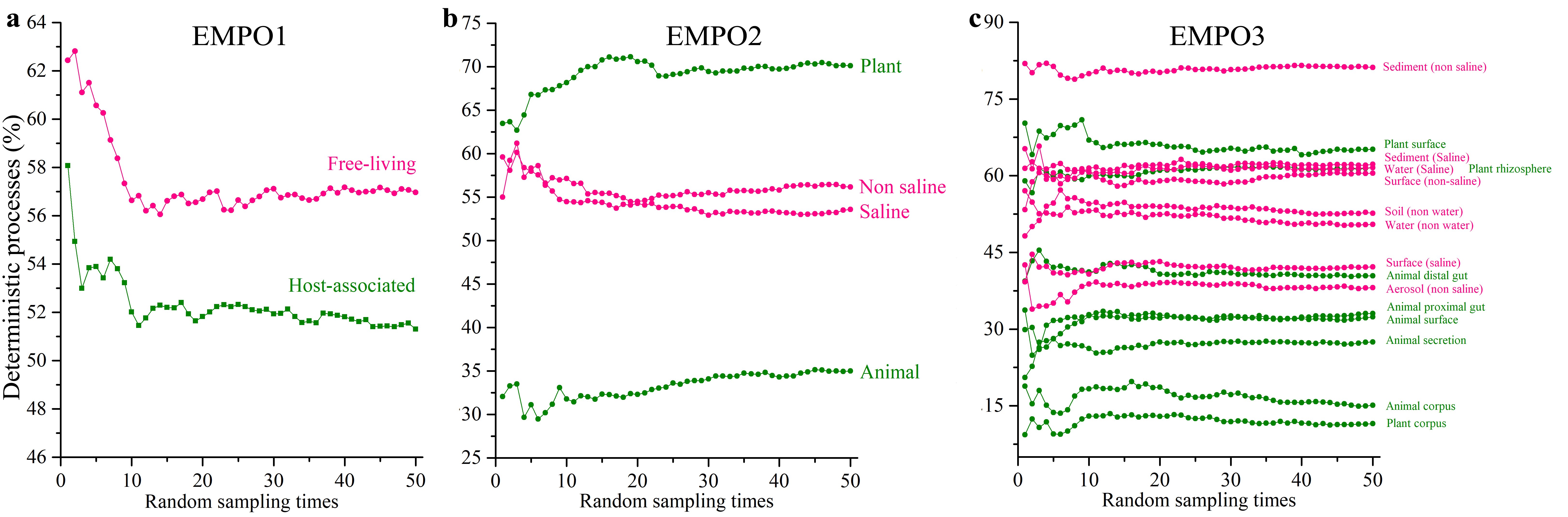

Supplement: Fig. S3 — Proportions of the deterministic processes for microbial community assembly in different environment types with the sampling times. a, EMPO1. b, EMPO2. c, EMPO3. Olive represents host- associated and magenta represents free-living. [file msystems.01289-22-s0003.tif]

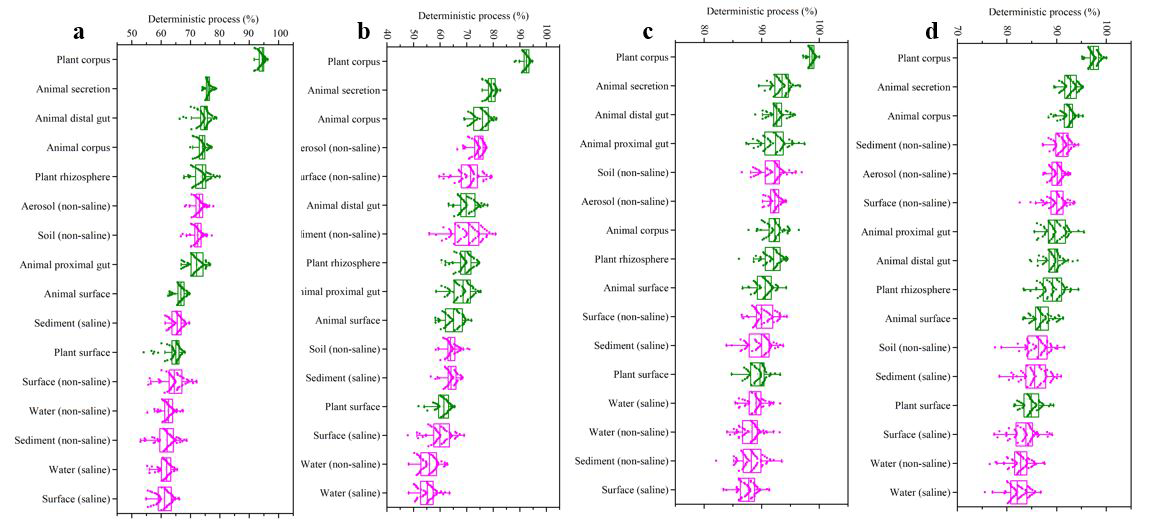

Supplement: Fig. S4 — The proportions of deterministic processes of microbial genes predicted using PICRUSt in different environment types (EMPO3). a, KEGG (unweighted model). b, COG (unweighted model). c, KEGG (weighted model). d, COG (weighted model). Each point represents the result of a single random sampling. For the box plots, the middle line indicates the median, the box represents the 25th–75th percentiles. Environment types were classified by EMPO, olive represents host-associated and magenta represents free-living. [file msystems.01289-22-s0004.tif]

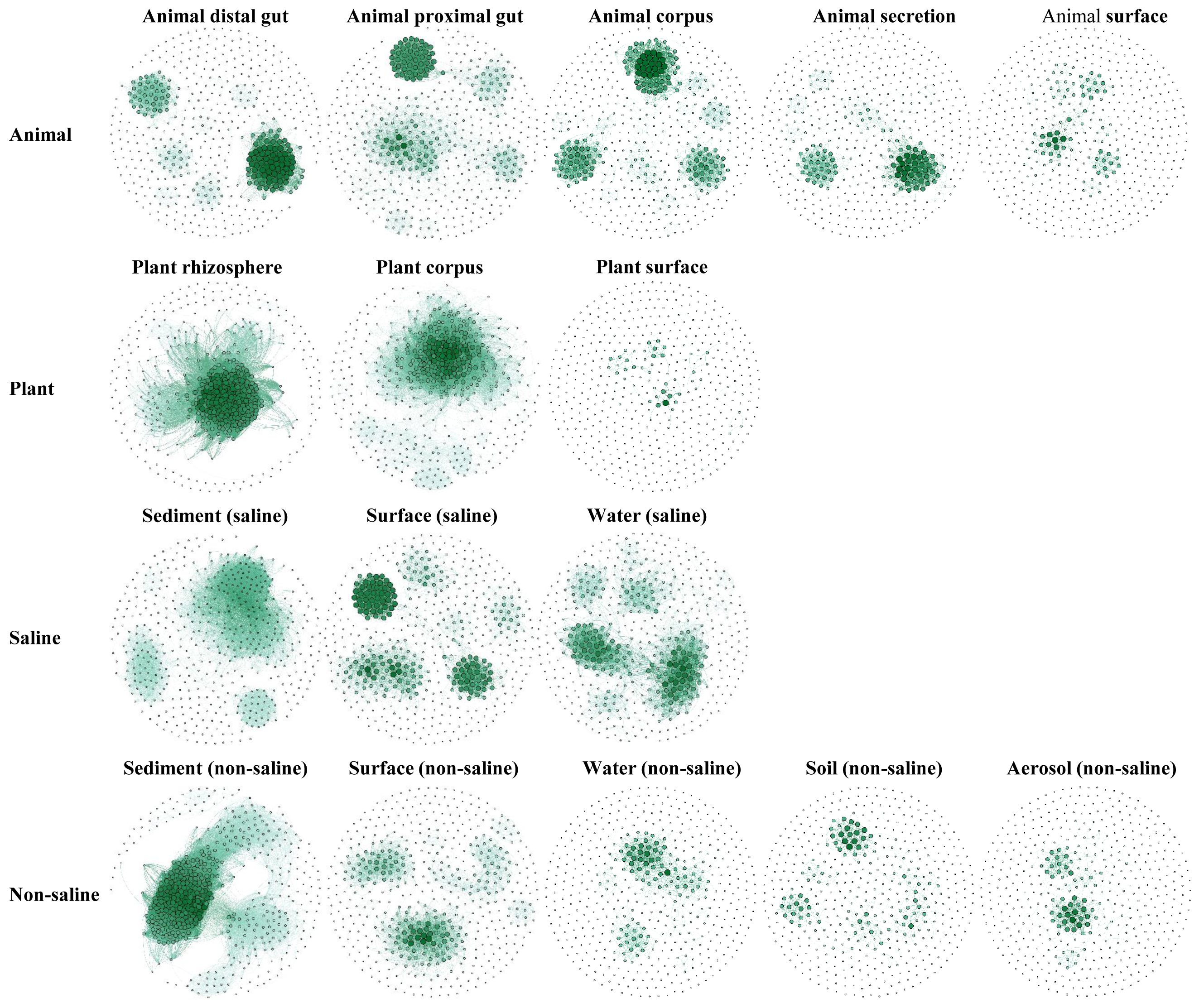

Supplement: Fig. S5 — Co-occurrence network analysis of the dominant bacterial ASVs (top 500) in different environment types (EMPO3). Each node represents an ASV, and the line means a significant correlation between the two ASVs (Spearman’s test, r > 0.5, p < 0.05). [file msystems.01289-22-s0005.tif]
